# Aesthetic refinements in 2nd toe-to-thumb transfer

**DOI:** 10.1186/1753-6561-9-S3-A63

**Published:** 2015-05-19

**Authors:** Zhao Jianyong

**Affiliations:** 1 Department of Hand Surgery, Cang-zhou Hospital of Hebei Medical University, Cang-zhou, China

## Objective

The hand is a body part which is constantly in view thus the second toe-to-thumb transfer needs to be aesthetically pleasing. Our study measures the absolute values of second toes, big toes, thumbs and their nails with regards to their width, length and circumference and analyses the ratios of between these relative values. With this anatomical data we intend to discover a way to enhance a more aesthetic second toe-to-thumb transfer surgery.

## Methods

The absolute values of the width (W), length (L) and circumference (C) of second toes, big toes, thumbs and their corresponding nail plate measurements were obtained from 40 normal Chinese adult (23 male, 17 female). Utilising statistical techniques (SPSS19.0), the values were computed at corresponding anatomical site to provide the anatomical data and to analyze these ratios between thumbs and toes (second toe and big toe). Based on the results of anatomical measurement, 20 cases which underwent thumb amputations were treated by way of our new aesthetic modification in toe-to-thumb transfer surgery.

## Results

There were significant differences between the ratios of L_1_/L_2_, W_1_/W_2_, C_1_/C_2_ of the second toes and the thumbs. Under the guidance of anatomical measurement, when the ratios of the transferred second toe were close to the ratios of the patients’ own healthy thumbs, a better aesthetic appearance was achieved.

## Conclusions

The absolute values of second toes, big toes, thumbs and their nail plates’ width, length and circumference, (L_1_/L_2_,W_1_/W_2_,C_1_/C_2_) in vivo are valuable supplement to the anatomical data. We attempt to adjust the transferred second toe’s relative key ratios and ensure the transferred second toe is of a closer physiological structure to the healthy thumb. The ratios of L_1_/L_2_,W_1_/W_2_,C_1_/C_2_ of the second toes and the thumbs is extremely useful and this novel approach provides patients with a more aesthetic appearance of the new thumb.

**Figure 1:**
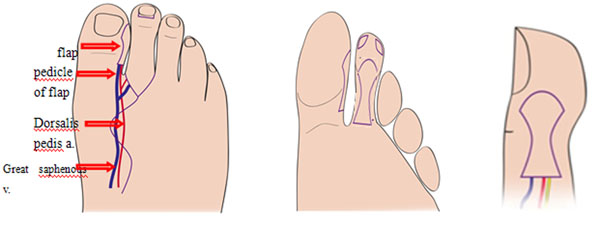
The aesthetic refinements in second toe-to-thumb transfer surgery

